# Observations on Dr. Harrison's Bill

**Published:** 1811-06

**Authors:** 


*To the Editors of the Medical and Physical Journal.*

GENTLEMEN,

THOUGH your Correspondent H. R. has professed to "reply in a few words" to the observations on what he is pleased to call (I will not say unjustly) "the uncourteous attack" on Dr. Harrison's Bill, which you did me the favour to insert in your Journal for March last, I cannot yet acknowledge myself satisfied fied with his explanations, or admit that he has obviated any of the material objections to the proposal in question. I must, indeed, plead guilty of some inaccuracy of language in describing the leading features of the Bill; but I was perfectly aware, that the comfort and advantage of "the present generation" were not consulted in its provisions; and instead of thinking it too *mild*, as H. R. would insinuate, I have spoken, in express terms, of "the *mischiefs* that must arrise" from its enactment, because "every thing seems to have been studiously contrived with the view of degrading all that is great, and learned, and respectable in the profession; of consolidating every abuse that exists, and of perpetuating every irregularity of which Dr. Harrison and his fellow-reformers so loudly complain." A bill to oblige all future physicians, surgeons, and apothecaries, "to take out annually certificates" of leave to practise, just in the same manner that people take out licences to kill game, or to retail beer!—a bill to enable half-educated country magistrates, or their clerks, to license bone-setters!—a bill which interferes not with the sale of stamped medicines!—Is this the mild "bill *for the improvement* of the Medical, Surgical, and Veterinary Sciences," which has been "*most favourably* received by the faculty," which will produce "the gradual amendment of medical men," and "be productive of immediate advantages to society at large?" No, ye sapient worthies of the Lincolnshire Benevolent Medical Society, this bill has been egregiously misnamed; it should be termed, A BILL FOR THE ENCOURAGEMENT OF IRREGULAR PRACTITIONERS, AND FOR PROMOTING THE SALE OF QUACK MEDICINES.

Your Correspondent, however, is not disposed to admit these consequences, and declares himself unable to see either how "additional countenance" will be afforded "to the sale of stamped medicines," or how "the powers of the existing corporations" will be strengthened by the passing of the projected act. Now, really, Gentlemen, as to the first point, I scarcely know how to express my astonishment that Dr. Harrison and his friends should, notwithstanding all the objections that have been urged, and notwithstanding all the happy ridicule of Dr. Beddoes on this great defect of their scheme, persist in asserting that it gives no sanction to the sale of quack-medicines. Cannot they be made to understand, that if, in any legislative measure professing to be "for the improvement" of a particular art, a saving clause be introduced in favour of any abuse prevalent in that art—cannot they be made to understand, that this abuse becomes, by such a proceeding, directly countenanced and legalized? Cannot they perceive how much it is the interest of the proprietors of *nervous cordials*, *solar tinctures*, and *vegetable balsams*, to assist them in forwarding a bill that shews them such

 marked marked favour? But the Association, it seems, discovered, that they were treading upon tender ground, when they touched upon the province of quackery, and deemed the clause in question necessary to assuage the fears of those alarmists who might apprehend a falling off in the revenue from the passing of the proposed act. Such an extreme degree of prudence may be commendable, but on the wisdom and policy of the proceeding it is impossible to bestow applause.—The assertion, that the powers of the existing medical corporations would be confirmed and increased by an act which greatly extends their jurisdiction, seems to require equally little proof. It is true "the Licentiates of the College of Physicians *may* prefer their claims with as much cogency, after, as before the passing of the Bill, as may the commonalty of surgeons and independent apothecaries, for the severe treatment received from their respective corporations:" but does your correspondent believe, that the vast augmentation of funds which those societies must experience, if the scheme were carried into effect, would be of no account; or that the disposition to abuse their authority would not be increased by it? Let him take the case of the London College of Physicians which has now on its list nearly three hundred members, and which after the passing of the proposed act, would probably reckon thousands; let him consider how its finances would be improved by this addition, and then say, whether its rulers would not be supplied with all the power which wealth could bring, "to purchase up submission and to overawe resistance."

The Association resolved, at one of their earlier meetings, "that the reform, *to be complete*, *must pervade all the departments in physic*;" and yet, according to their present plan, the most perfect immunity would be granted to all the *intruders* "at present practising, however irregularly." They regret, "that women, porters, grinders of paint, pestle-pounders, distillers of peppermint, &c. soon think themselves qualified to sell drugs, and prescribe them too, in all cases;"* and yet they introduce a clause into their bill, by which these anomalous beings are to be protected, and empowered to prescribe and kill "*impune per totam terram*." It may be urged, that the magistrates, who are to take cognizance of their behaviour, will be able to check any daring attempts at imposture, or, to use the words of your correspondent, "secure the suppression of notorious offenders." *Sed quis custodiet ipsos custodes?*—Will those guardians of the public health be so far above all suspicion themselves, that the public may safely rely on their vigilance? Will they be always so well versed in medical science, as to

* Harrison's Address, p. 67.

judge judge correctly of medical ability? And will they never bestow licences but upon those who are truly skilled in the art?—To these questions the best answers will be found in the attestations of wonderful cures performed by advertising quacks, which are daily presented to the world, under the hands of worshipful judges, and right reverend bishops; who, whether it be in their own cases, or in those of their butlers and coachmen, decide on the virtues of a medicine with much greater confidence than Sydenham or Ratcliff would have shewn; and who declare, without scruple, their implicit belief in the efficacy of *nostrums*, which are commended for the cure of all incurable diseases. When such is the case, what man in his senses would think of appealing to Their Worships the Justices at the General or Petty Sessions, as a competent tribunal to decide on the practice of physic?

In perusing the "Address to the Lincolnshire Society," I was not a little surprised to find the author alluding to the late Dr. Beddoes, as one of those who were disposed to second their view—to the same Dr. Beddoes, who, in his "Letter to Sir Joseph Banks," had so successfully exposed the futility of their former scheme, and who, in the following passage seems to have anticipated something like their present bill.

"To an individual, or a body, which has once lost the public confidence in a matter dependent upon skill, *rehabilitation* by dry statute is impossible. The power of a former French king, or present French emperor, would be unavailing. Whereas, to use, by way of example, the words of the Association, '*abundant proofs have been produced of the deplorable state of society, in being exposed to the injuries resulting from a numerous race of unqualified practitioners, and the consequent discouragement of well-educated members of the faculty*,' what would it avail, if, to this litany, our legislators were, *nemine contradicente*, to respond—'*be it therefore enacted, by and with* ———, *that Doctors of Medicine, of the Universities A. B. C. are from the present date respectable; and let them henceforth be respected, and feed in preference to all pretenders whatever?*" (Letter, p. 16.)

"There are laws," says Montesquieu, "so little understood by the legislator, as to be contrary to the very end he proposed;" and of exactly this nature would be those which our medical reformers are desirous to enforce. Nothing, in fact, can be more preposterous, than the idea of regulating the practice of physic by an act of parliament, at least by such an act of parliament as that now recommended. If carried into execution, it would have about the same effect, and be about as much regarded by the public as the ordonnance which was issued by the Spanish court in the year 1785, when a destructive epidemic raged at Carthagena, gena, directing the physicians of that place to administer no other medicine to the sick than the opiate of Don Joseph Masdeval—subjecting them to the Intendant of the Dock-yard—and commanding them to prescribe according to his instructions. The physicians, after some fruitless expostulations, found themselves compelled to obey; but the people were more refractory, and treated the royal mandate with such disrespect, that rather than submit to the prescription of the opiate, they actually refused to send for any medical assistance whatever. At length, the Court, finding that the physicians were starving, while the citizens continued to die in great numbers, agreed to compromise the matter, by confining the operation of the obnoxious edict to the Royal Hospital*.

Yet we are told, that the Bill "has been most favourably received by the faculty†." By what part of the faculty, I would ask, has it been thus "favourably received?" On a point of such magnitude why not give the names? My opportunities of learning the sentiments of my brethren are probably as great as those which Dr. Harrison enjoys; and yet, upon no occasion when his plan has been the subject of discussion, have I heard a single voice raised in its praise. None of the Reviews have had the complaisance to applaud it; and the Colleges who were applied to by the Secretary of State for their sentiments on the subject, are, as I have access to know, unanimous in its condemnation.

On the whole, I must give it as my opinion that if the former scheme of the Association was futile, their present one is downright absurd, or, rather that it is calculated to extend the mischiefs it was intended to remedy; that there is scarcely any one irregularity in medical practice which would not find a fostering provision in its enactments, and that there is not one excrescence of the profession which it would deaden or destroy. I have examined it in all its parts with the most scrupulous attention; I have viewed it in its probable consequences; I can perceive nothing in it but the seeds of disorder and confusion; and till I read the pamphlet in which it is detailed, I could not have believed that after the many facilities which had been afforded to the views of the reformers, after the ample discussion which their measures had undergone, and after the many useful hints which had been furnished them, I could not have believed that it was possible for any set of men so circumstanced to have toiled so long and to have done so little; to have conceived with such mighty efforts, and to have produced nothing but a miserable abortion.

* Townsend's Journey through Spain, Vol. III. page 137-142.

† Harrison's Address.

Before

Before I conclude, allow me to request H. R. to explain the data on which he grounds his very singular calculation, curtailing the "aggregate lives" of the medical men of the United Kingdom to "seven years' purchase" and killing them off at the rate of 2000 a year. Though, perhaps, but few of the votaries of Esculapius now reach a Hippocratic old age, yet a considerable proportion of them contrive, I believe, to spin out their existence to *ten times seven years*, if not to the advantage of their patients, at least to the manifest annoyance and mortification of their more youthful competitors.

I remain, Gentlemen,

Your most obedient Servant,

*London, May* 13. A. H.

